# Scattering properties and internal structure of magnetic filament brushes

**DOI:** 10.1039/c6sm02606k

**Published:** 2017-03-13

**Authors:** Elena S. Pyanzina, Pedro A. Sánchez, Joan J. Cerdà, Tomàs Sintes, Sofia S. Kantorovich

**Affiliations:** a Ural Federal University , Lenin av. 51 , 620000 Ekaterinburg , Russia . Email: elena.pyanzina@urfu.ru; b University of Vienna , Sensengasse 8 , 1090 Vienna , Austria; c Instituto de Física Interdisciplinar y Sistemas Complejos (CSIC-UIB) , E-07122 Palma de Mallorca , Spain

## Abstract

Practical applications of polymer brush-like systems rely on a clear understanding of their internal structure. In the case of magnetic nanoparticle filament brushes, the competition between bonding and nonbonding interactions—including long range magnetic dipole–dipole interactions—makes the microstructure of these polymer brush-like systems rather complex. On the other hand, the same interactions open up the possibility to manipulate the meso- and macroscopic responses of these systems by applying external magnetic fields or by changing the background temperature. In this study, we put forward an approach to extract information about the internal structure of a magnetic filament brush from scattering experiments. Our method is based on the mapping of the scattering profiles to the information about the internal equilibrium configurations of the brushes obtained from computer simulations. We show that the structure of the magnetic filament brush is strongly anisotropic in the direction perpendicular to the grafting surface, especially at low temperatures and external fields. This makes slice-by-slice scattering measurements a technique very useful for the study of such systems.

## Abstract 2

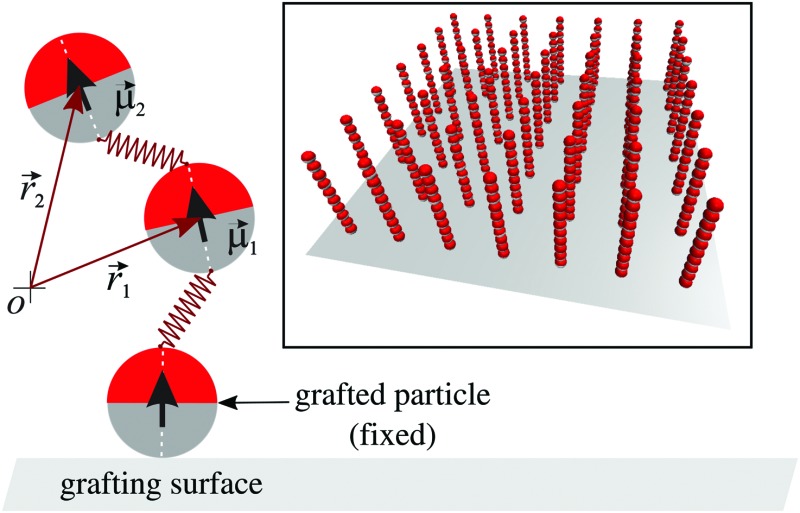
We predict by means of computer simulations the in-plane scattering response of magnetic nanoparticle filament brushes, mapping it to their internal detailed structure.

## 1 Introduction

Stimuli-responsive materials based on polymers are one of the main research topics in soft matter physics.^1–3^ Among the different approaches to design such ‘smart’ materials, polymer brush structures—*i.e.*, relatively dense arrays of polymer chains grafted by one of their ends to a substrate—^4–6^ are broadly used to create stimuli-responsive coatings and surfaces with a wide spectrum of applications.^7–9^ Frequently, the responsiveness of these coatings is provided directly by the polymers that form the brush. In recent years, it has become possible to synthesise polymers that are sensitive to many different stimuli, like temperature and electromagnetic radiation,^10^ pH,^11^ ionic strength,^12^ specific additives and substances,^13^ or external fields.^14^ The use of electric or magnetic fields as stimuli is very appealing for technological applications due to the fast and accurate control that is achievable over their intensity and spatial distribution. The combination of these interesting properties with the broad availability of polyelectrolyte substances—ubiquitous in many biochemical systems—has largely stimulated research efforts devoted to studying the response of polyelectrolyte brushes to external electric fields.^15–17^ However, the inexistence of known polymer substances with pronounced magnetic properties—except for the case of a few compounds at very low temperatures—^18–20^ necessitates the combination of polymers with other materials—usually solid magnetic micro- or nanoparticles—in order to create soft magnetoresponsive composite systems.^21^ In spite of the added complexity in the synthesis of these materials, the use of magnetic fields as control stimuli has a specific advantage over other parameters: the magnetic field has effects only on the elements of the system that are responsive by design—*i.e.*, the magnetic particles—avoiding undesired side effects on any other part. This is particularly convenient for surfaces designed to interact with biochemical substances and other soft materials, which in most cases are very sensitive to several of the aforementioned stimuli.

To the best of our knowledge, only a few approaches have been explored to date in order to create surfaces of magnetoresponsive polymer composites. Interesting examples are the field-induced assembly of polymer-coated magnetic particles into dense arrays of nonpermanent linear chains, adopting a transient polymer brush-like structure on a surface,^22^ or the direct embedding of magnetic nanoparticles into actual polymer brushes.^23^ In such approaches, unfortunately, the control of the internal structure of the brush is rather limited. Recently, we proposed an alternative strategy to create magnetoresponsive polymer brush-like structures that is intended to provide high responsiveness and facilitate the accurate control of the properties of the coating. This strategy is based on the use of pre-assembled linear chains of magnetic nanoparticles, stabilised with polymer crosslinkers, to form the magnetic brush.

Permanent chains of polymer crosslinked magnetic particles, usually known as magnetic filaments, were synthesised for the first time more than one decade ago with the purpose to work as magnetically driven microfluidic propellers.^24^ In recent years, these magnetic chains have found a growing range of applications,^25^ in parallel with the development of the synthesis techniques. Nowadays, it is possible to synthesize filaments of magnetic microparticles, with high control over the flexibility of the filament backbone, that behave like a semiflexible polymer chain.^26^ Most of the attempts to create one-dimensional chains of crosslinked nanoparticles have provided so far very stiff filaments.^27–30^ Semiflexible fibre structures of magnetic nanoparticles have been obtained instead by crosslinking them in bunches.^31^ However, we expect that other approaches to the synthesis of magnetic filaments, like the *in situ* mineralization of the magnetic nanoparticles along the backbone of polymer strands,^32,33^ or the application of cutting-edge experimental techniques, like DNA designed self-assembly methods,^34,35^ will soon provide nanoparticle filaments with highly tuneable magnetic and mechanical properties. To the best of our knowledge, magnetic filaments have not yet been used to create experimental polymer brush-like dense arrangements, but we are convinced that their potential makes experimental efforts in this direction worthwhile, and we expect the predictions we provide in our theoretical studies to stimulate the interest of experimentalists. Inspired by this perspective, we started to analyse theoretically the properties of a model system of magnetic nanoparticle filaments arranged into a polymer brush-like structure, as a promising design for a magnetoresponsive coating.^36,37^

In our previous studies, we showed that the magnetic filament brush has a more pronounced structural change between equilibrium conformations as a response to changes in the background temperature and/or external magnetic fields perpendicular to the grafting surface than analogous polyelectrolyte brushes with extended electric dipoles or embedded magnetic particles,^36^ as a consequence of the more compact structure of the magnetic brush at zero field and the absence of screening of the magnetic interactions. We also showed that this structural behaviour is determined by the characteristic self-assembly of the magnetic particles that form the filaments, which is driven by dipole–dipole interactions and their interplay with the thermal fluctuations and constraints associated with the brush structure.^36,37^ At this point, it is crucial to complete the study of the internal structure of the brush in thermodynamic equilibrium—as it determines the macroscopic response of the system—and to establish how this internal structure can be connected to experimental observables. In particular, here we aim to predict from simulation data the experimental measurements that one can expect for the equilibrium structure of magnetic filament brushes.

Scattering techniques are one of the main experimental tools currently available to study the internal structure and self-assembly properties of systems formed by magnetic nanoparticles,^38–43^ and as such will be essential in the upcoming experimental development of the system we study here. Certain scattering techniques developed recently for the characterisation of interfaces, like the grazing incidence small angle X-ray and neutron scattering methods (GISAXS and GISANS, respectively) open up the possibility to obtain information on the structural organisation of the system in a plane parallel to the interface.^44–52^ This spatial discrimination can be very useful to study systems with a complex and/or strongly anisotropic internal structure, as is expected to be the case of magnetic filament brushes. However, even the most advanced scattering techniques tend to provide rather smoothened structural information. This makes the measurements performed on strongly anisotropic systems particularly difficult to analyse. For this reason, thorough theoretical modelling of the detailed internal structure of magnetic filament brushes and its connection to the scattering properties of these systems is essential. Taking a first step in establishing this connection is the main goal of this work.

The paper is organised as follows: in Section 2 we introduce the model and describe the simulation approach. After that, we split our Results and discussion (Section 3) into two parts: in Section 3.1, we start with calculating the scattering intensities and structure factors measured in a plane parallel to the grafting surface for the simulated equilibrium configurations, first for the whole brush and, second, for a slice-by-slice partitioning of the system. In Section 3.2 we focus on the characterisation of the formation of close contact particle pairs and the interpretation of the finest details of the slice-by-slice scattering measurements. Finally, a summary of our study is provided in Section 4.

## 2 Brush model and simulation approach

Our theoretical study on magnetic filament brushes is based on a coarse-grained phenomenological model of the filaments and their polymer brush-like arrangement.^36,53^ The conditions of the experimental system that this model is intended to represent are the following: we assume the magnetic filaments to consist of one-dimensional chains of permanently crosslinked ferromagnetic nanoparticles. These particles are spherical and monodisperse, with a permanent magnetic moment that is fixed with respect to their solid body structure and, as is usual in magnetic nanoparticles, a polymer coating that stabilises them in the carrier fluid. Some polymers from the coating work as crosslinkers by binding their ends to the surfaces of neighbouring particles in the filament. The crosslinking is assumed to be established under field assisted assembly of the nanoparticles into straight chain configurations, with their magnetic moments co-aligned into a head-to-tail arrangement. This means that deviations from the head-to-tail alignment of two crosslinked particles will produce a stretching of the polymer crosslinkers and, therefore, will have an energy penalisation. Finally, these filaments are arranged into a brush structure by densely grafting them to an inert steric substrate with a flat surface. Such grafting is assumed to be achieved by means of strong binding of the particle at the grafted end of each filament, so that such particles remain immobile on the substrate.

**Fig. 1:**
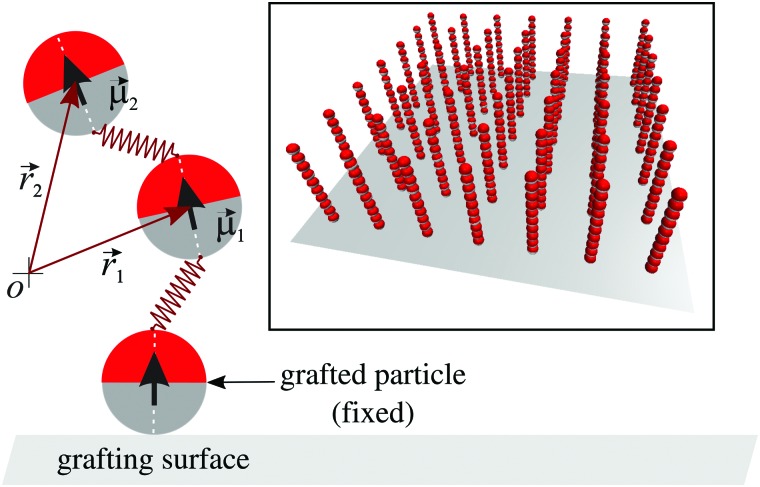
Scheme of our model for a ferromagnetic nanoparticle filament grafted to a flat surface (main figure) and nonequilibrated configuration of a model brush system formed by filaments of length *N* = 10 particles and grafting density *σ* = 0.040 (inset).

The coarse-grained representation of the system described above, already introduced in our previous studies,^36,37^ uses a bead-spring modelling approach for the magnetic filaments. Briefly, the ferromagnetic nanoparticles are modelled as soft core spherical beads with a given characteristic diameter, *d*. Their magnetic moments are described by a point magnetic dipole, *μ⃑*, located at the centre of each sphere. Therefore, there is a long-range dipole–dipole interaction between any pair of beads *i* and *j*:1
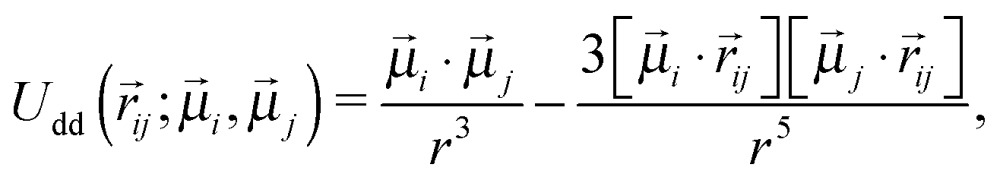
where *μ⃑*_*i*_ and *μ⃑*_*j*_ are the dipole moments corresponding to each bead, *r*_*ij*_ = *r*_*i*_ – *r*_*j*_ is the displacement vector connecting their centres and *r* = ∥*r*_*ij*_∥ is the modulus of such a vector. The soft core interaction between the beads that mimics the effect of the polymer coating of the nanoparticles is given by the Weeks–Chandler–Andersen pair potential (WCA),^54^
2
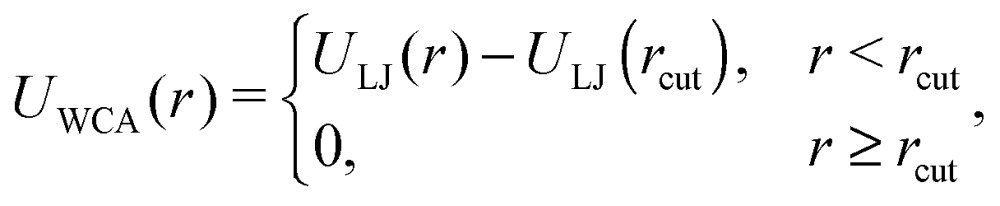
where *U*_LJ_(*r*) is the conventional Lennard-Jones potential (LJ),

3 *U*_LJ_(*r*) = 4*ε*_s_[(*d*/*r*)^12^ – (*d*/*r*)^6^],

which in expression (2) has been truncated at the position of its minimum, *r*_cut_ = 2^1/6^ *d*, and shifted by its corresponding depth, *U*_LJ_(*r*_cut_), to make the resulting potential purely repulsive. The bonding effects of the crosslinkers are modelled by means of a simple harmonic spring whose ends are attached to the surface of the crosslinked beads. The spring attachment points are located in each bead at the projection points of the head and the tail of the dipole moment associated with the bead, at a distance *d*/2 from its centre. The corresponding bonding potential for two crosslinked particles *i* and *j* is:^53,55^
4
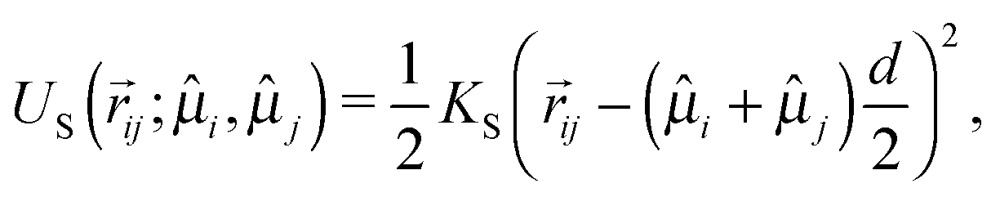
where *μ̂*_*i*_ = *μ⃑*_*i*_/∥*μ⃑*_*i*_∥ and *μ̂*_*j*_ = *μ⃑*_*j*_/∥*μ⃑*_*j*_∥ are the unitary vectors parallel to each associated dipole moment. This bonding potential, whose bead-spring representation is shown schematically in Fig. 1, introduces a coupling between the orientation of the magnetic dipoles and the filament backbone. The repulsive steric interaction between the filament beads and the grafting surface, which corresponds to the plane *z* = 0, is given by a truncated shifted 9-3 LJ potential.^56^ This potential is the result of, first, integrating the LJ potential (3) over an infinite flat surface, which gives5
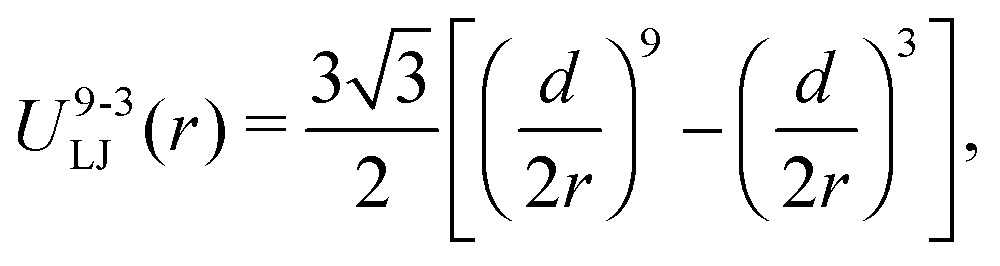
where *r* is the *z* coordinate position of the bead; this expression is then truncated and shifted in the way defined in eqn (2), using its corresponding minimum for the cutoff, *r*_cut_ = 3^1/6^ *d*/2. Finally, a model brush structure is obtained by grafting to this surface a set of identical filaments, each one formed by a chain of *N* beads with bonds between every pair of first nearest neighbours. The grafting is mimicked by simply fixing the orientation and the position of the center of the bead corresponding to the grafted end of each filament. For simplicity, the grafting positions are placed on a square lattice arrangement in the *x*–*y* plane, with a separation constant *a* and a fixed vertical position *z* = *r*_cut_ = 3^1/6^ *d*/2. Therefore, the number grafting density of the brush, *σ*, that is defined as the number of grafting positions per unit of surface area, is *σ* = *a* ^–2^. The magnetic dipoles of the grafted beads are set to point permanently in the *z* direction. The rest of the beads of each filament are initially placed according to a perfect head-to-tail arrangement, also pointing parallel to the *z* direction and with a separation *d* between first nearest neighbours. The inset in Fig. 1 shows an example of the initial configuration of the filaments forming a brush with a relatively low grafting density.

The model described above was used to study the equilibrium behaviour of a magnetic filament brush at different temperatures and under external fields by means of extensive off-lattice computer simulations in the canonical ensemble. The simulation method was molecular dynamics with a Langevin thermostat, which treats implicitly the effects of the thermal fluctuations of the background fluid.^57^ The brush was placed into a cubic simulation box of side length *L* ≫ *Nd* without any spatial discretisation. In order to mimic a pseudo-infinite system, lateral periodic boundaries were imposed and the dipolar-P^3^M^58^ and dipolar layer correction^59^ algorithms were used to calculate the long-range magnetic interactions. Details of these algorithms and the simulation protocol can be found in our previous studies^36,37^ and references therein.^58–62^ The simulations were performed using the ESPResSo 3.2.0 package.^63,64^

In the following, we measure all the physical parameters of the system in reduced units, taking as reference the reduced characteristic diameter and mass of the magnetic nanoparticles, *d* = 1 and *m* = 1, respectively, and the prefactor of the reduced soft core potential (2), *ε*_s_ = 1. The values taken for the interparticle interaction parameters are *K*_s_ = 30 for the prefactor of the potential (4) and *μ* ^2^ = (*μ⃑*·*μ⃑*) = 5 for the squared dipole moment of the magnetic beads. The first parameter gives average bond lengths close to the characteristic diameter of the bead soft core, *d* = 1, and a maximum distance between two bonded particle surfaces not larger than half of such diameter. These choices correspond approximately to, for example, magnetite spherical particles with a magnetic core diameter of approximately 25–30 nm and a 5–10 nm polymer coating, which is also used to crosslink the particles. Since the main factor determining the self-assembly behaviour of magnetic nanoparticles is the ratio between the strength of the dipole–dipole pair energy and thermal fluctuations—and not their absolute values—moderate changes of the dipole moment of the particles in our simulations are not expected to produce qualitatively different results, but only a shift in the response of the system to temperature and external fields. For large changes in the value of *μ*, qualitative differences should be expected due to the distinct impact of magnetic interactions and thermal fluctuations on the chain configurational entropy of the filaments.

## 3 Results and discussion

The results we present here correspond to the simulation of brushes made of magnetic filaments with a length of *N* = 10 nanoparticles. Two grafting densities have been sampled, *σ* = 0.040 and *σ* = 0.111, using simulation boxes of side lengths *L* = 35 and *L* = 30, respectively, which corresponds to at least three times the contour length of the filaments. We study the behaviour of these brushes for different reduced temperatures in a range *T* ∈ [0.35,5.0]. We also investigate the effect of an external magnetic field *H* ∈ [0.5,4.0].

### 3.1 Scattering properties

The first point we want to clarify here is whether it is possible to measure by means of scattering experiments the changes in the internal structure of a magnetic filament brush, particularly the changes that this system is expected to exhibit—according to our previous studies—^36,37^ as a response to variations in the background temperature and the application of an external magnetic field perpendicular to the grafting surface. The anisotropy of this system dictates the need to analyse the scattering patterns in the horizontal and vertical directions separately. However, scattering measurements in the vertical direction are expected to be much more difficult to perform accurately than the ones in the horizontal plane. For this reason, here we will focus only on the latter.

Any scattering experiment provides as an outcome two-dimensional intensity maps of the scattered beam. Such intensities obey the general expression

6 *I*(*q*) = *F*(*q*)*ρS*(*q*)

where *F*(*q*) is a form factor that, for a system of monodisperse spheres with radius *R* = *d*/2, is simply given by^65^ *F*(*q*) = (3[sin(|*q*|*R*) – |*q*|*R* cos(|*q*|*R*)]/(*qR*)^3^)^2^, *ρ* is the density and *S*(*q*) is the structure factor, a function that includes the information about the internal structure of the system in the Fourier reciprocal space.^65^ The vector *q* denotes the difference between the incoming and outcoming beam wave vectors. From simulations, one can obtain the horizontal component of *S*(*q*), *S*(*q*_*xy*_), as:7
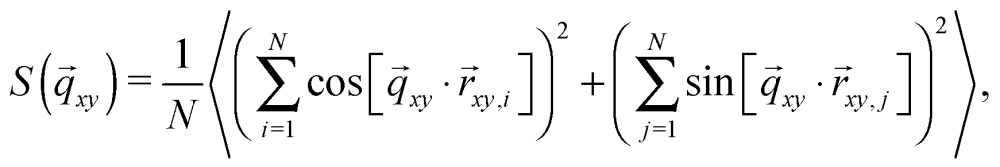
This expression sums up the contributions of all *N* particles in the system, whose positions are given by *r*_*i*_. The horizontal component of the vector *q* is *q*_*xy*_ = (*q*_*x*_,*q*_*y*_,0) = (*l*,*m*,0)2π/*L*, where *l* and *m* are integer numbers. The average · is performed over measured configurations.

**Fig. 2:**
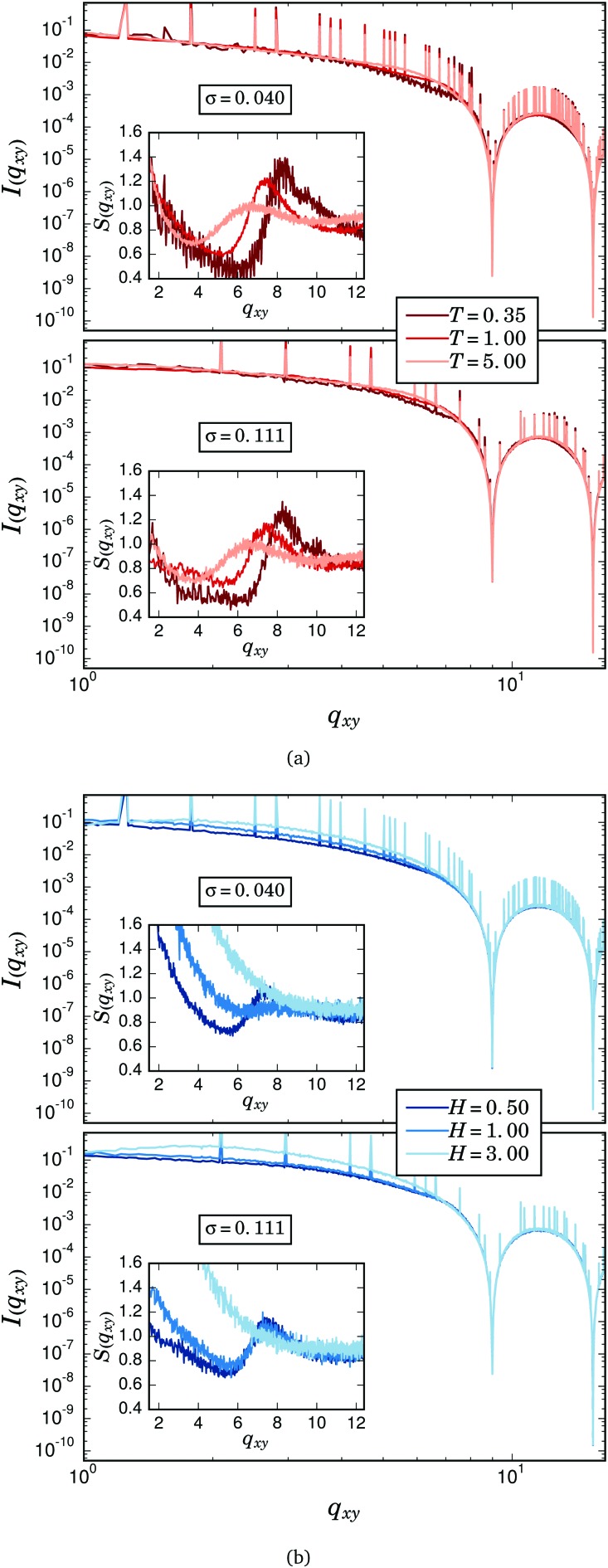
Intensity profiles (main figures) and structure factors (insets) calculated along the horizontal direction for both analysed grafting densities. Sharp peaks corresponding to the square lattice arrangement of the grafting points have been removed from *S*(*q*_*xy*_) for clarity. (a) For different values of *T*. (b) For different values of *H* and *T* = 1.

According to eqn (6) and (7), we computed the intensity and structure factor as a function of the horizontal component of the wavevector, *I*(*q*_*xy*_) and *S*(*q*_*xy*_), for the whole equilibrium structures of the filament brushes obtained from our simulations. Fig. 2(a) shows the values of these functions for three selected temperatures and the two studied grafting densities. Sharp vertical peaks in the intensity profile are the signature of the square lattice arrangement used for the positions of the filament grafted ends.^66^ Regarding the effects of temperature, the height of these peaks decreases slightly with *T* due to the increase of the structural entropy of the filaments near to their grafting points. For both grafting densities one can barely see significant differences in the main intensity profiles for low and high *q*_*xy*_ values, whereas for intermediate values there is a nontrivial shift in the intensity with *T*. The structure factor, shown in the insets, clarifies this effect. The contribution from the square lattice to this latter parameter has been removed to ease the visualisation. It can be seen that *S*(*q*_*xy*_) has a clear local maximum at values of *q*_*xy*_ around 2π/*d* ≈ 2π, corresponding to the average centre-to-centre distance of particles in close contact. The position of this first maximum shifts slightly towards higher values of *q*_*xy*_ as the temperature is decreased due to the soft core nature of their steric repulsion: as thermal fluctuations decrease, particles in close contact tend to have on average a more favourable alignment of their dipoles, thus they experience a stronger dipolar attraction that pushes further against their soft core barriers and decreases their centre-to-centre distance.^37,67^ The height of the maximum also increases as the temperature decreases, indicating a higher amount of particles in close contact in the horizontal plane. This is the consequence of the characteristic low energy structure of the magnetic filament brush system: at low temperatures, the filaments tend to experience a strong bending that reduces the overall dipolar energy by connecting their free ends to the grafted ones of neighbouring chains.^37^ Filaments with such bended configurations keep many of their central particles in linear arrangements that are mainly parallel to the grafting surface, increasing significantly the number of close contact pairs measured by *S*(*q*_*xy*_). However, filaments at high temperatures adopt more diverse configurations and both horizontal segments of filaments and pure self-assembled horizontal pairs of particles are less likely within the brush. This contribution from bended filaments to the first maximum of the horizontal structure factor will be discussed in more detail below. Finally, the effects of the grafting density are much less pronounced: the signature of the square lattice is less visible for the higher *σ* and differences in the depths of the minima of the intensity profiles can also be observed depending on *σ*. The change of the grafting density mainly affects the low *q*_*xy*_ behaviour of the structure factor, not changing qualitatively the temperature dependent shape of its first maxima.

Fig. 2(b) corresponds to the same measurements of *I*(*q*_*xy*_) and *S*(*q*_*xy*_) for the case *T* = 1 and the presence of an external magnetic field of different strengths applied in the *z* direction. The impact of the field on the main profile of the intensity and the structure factor is very strong, particularly at low *q*_*xy*_. For the lower grafting density, at a field of strength *H* = 1, the first maximum of the structure factor almost vanishes, revealing in this way the absence of touching particles in the horizontal direction. This is due to the strong vertical straightening of the filaments that is induced at low grafting densities by relatively weak fields.^37^ For *σ* = 0.111, however, the filaments are in general much more entangled. This makes it necessary to apply stronger fields to obtain the same degree of vertical straightening of the chains,^37^ thus the existence of clear first maxima in *S*(*q*_*xy*_) persists at higher fields.

**Fig. 3:**
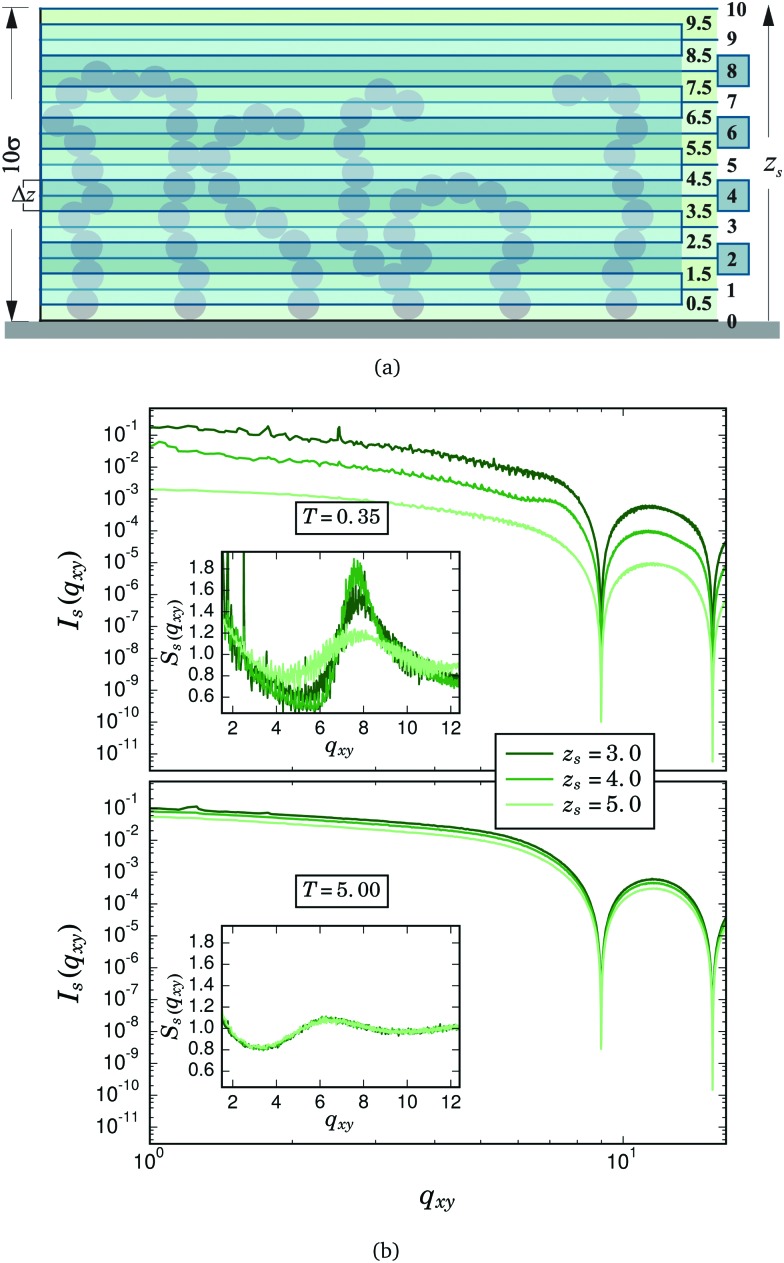
Scheme of the horizontal slicing system used to compute the local intensity and structure factor profiles in the horizontal direction, *I*_s_(*q*_*xy*_) and *S*_s_(*q*_*xy*_), as a function of the distance to the grafting surface, *z*_s_. (b) Examples of measured *I*_s_(*q*_*xy*_) (main figures) and *S*_s_(*q*_*xy*_) (insets), obtained for *σ* = 0.040, three selected slices (*z*_s_ = {3,4,5}), and two selected temperatures: *T* = 0.35 (upper panel) and *T* = 5.0 (lower panel). The contribution of the grafting square lattice to *S*_s_(*q*_*xy*_) has been removed for clarity.

Even though the scattering measurements clearly evidence the existence of a significant amount of touching particle pairs in the *xy* direction, especially at low temperatures and zero field, they do not shed any light either on the origin of these particle contacts, or on their exact location within the brush. Fortunately, the advanced scattering techniques mentioned above opened the possibility to perform scattering experiments that provide the structural information corresponding to a plane at a given distance from the grafting surface.^44–52,68,69^ Inspired by these experimental techniques, we decided to obtain analogous measurements from our simulation data. With this purpose, we computed the local values of *I*(*q*_*xy*_) and *S*(*q*_*xy*_) at a given distance from the grafting surface by splitting the system into a set of overlapping thin slices along the *z* direction, as shown schematically in Fig. 3(a). Each slice covers horizontally the whole simulation box, has a height Δ*z* = 1 and is characterised by the vertical position of its geometrical centre, *z*_s_. In this way, for a given *z*_s_, the sum in eqn (7) only considers the contributions from the particles whose vertical position, *r*_z_, satisfies *z*_s_ – Δ*z*/2 ≤ *r*_*z*_ ≤ *z*_s_ + Δ*z*/2. Fig. 3(b) shows several examples of the local values of *I*(*q*_*xy*_) and *S*(*q*_*xy*_) calculated with such a criterion for different *z*_s_, *σ* = 0.040, zero field and two selected temperatures. These in-plane local values of intensity and structure factor are denoted as *I*_s_(*q*_*xy*_) and *S*_s_(*q*_*xy*_), respectively. The upper panel of the figure corresponds to the lowest sampled temperature, *T* = 0.35. We can observe that *I*_s_(*q*_*xy*_) decreases significantly as one moves away from the grafting surface. This is a consequence of the fast decay of the density of particles with height that this system exhibits at low temperatures.^36^ In the inset we can see that the first maximum of the local structure factor depends on the vertical position of the slice in a nontrivial way: whereas its position does not depend on *z*_s_, its height changes nonmonotonically, signaling a higher number of horizontal close contact pairs at intermediate distances from the grafting surface. The lower panel, corresponding to *T* = 5, shows that at high temperatures the differences between slices tend to disappear.

**Fig. 4:**
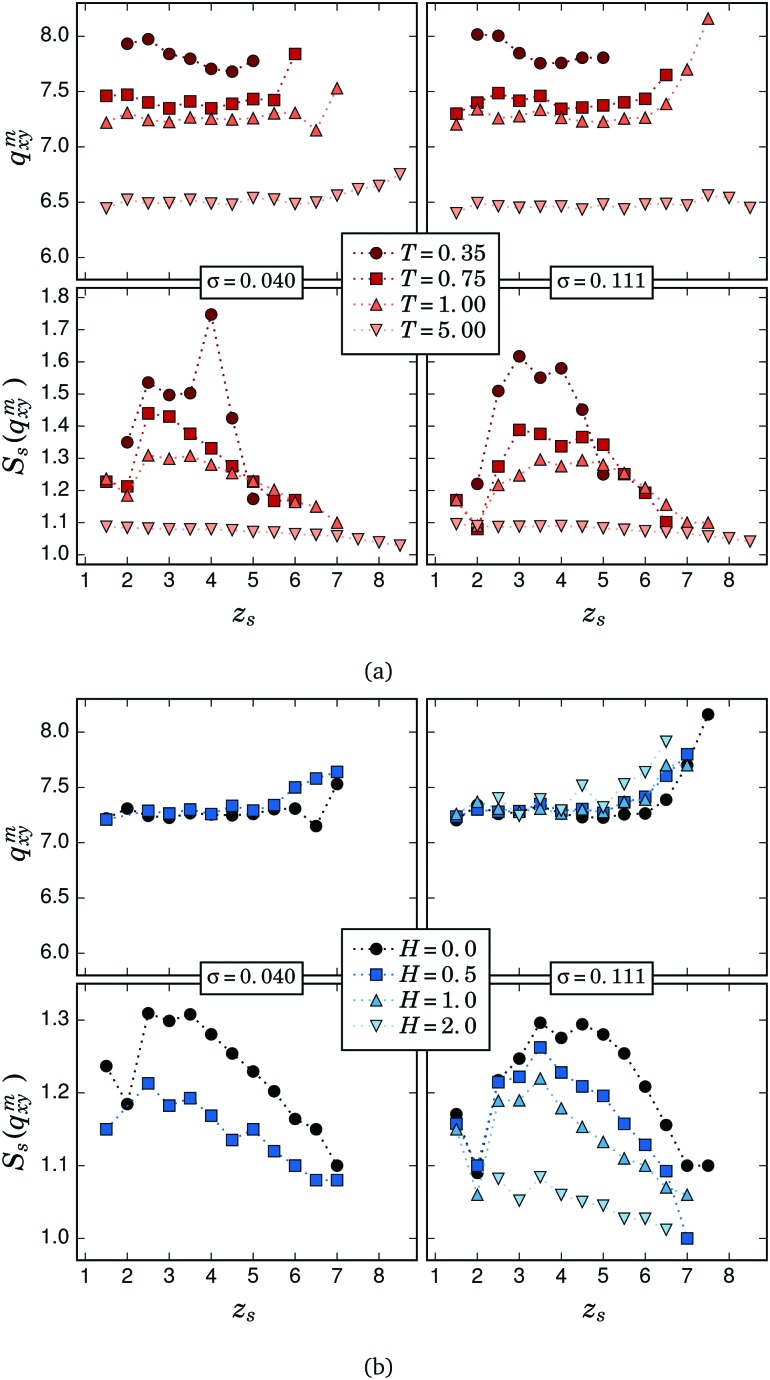
Position, *q* *m* *xy*, and height, *S*_s_(*q* *m* *xy*), of the first maximum of the local in-plane structure factor, *S*_s_(*q*_*xy*_), as a function of the distance to the grafting surface, *z*_s_. Missing points correspond to the absence of a clear maximum. (a) Results for different temperatures and zero field. (b) Results for different field strengths and *T* = 1.

In order to better analyse the results of the slice-by-slice scattering measurements, we focus on the dependence of the position, *q* *m* *xy*, and height, *S*_s_(*q* *m* *xy*), of the first maximum of the local structure factor on the system parameters. In Fig. 4 we plot *q* *m* *xy* and *S*_s_(*q* *m* *xy*) as a function of the distance to the grafting surface, *z*_s_, for both grafting densities and different temperatures (Fig. 4(a)), and fields (Fig. 4(b)). The values of *q* *m* *xy* and *S*_s_(*q* *m* *xy*) were determined from a cubic spline fit around *q*_*xy*_ ≈ 2π of the measured *S*_s_(*q*_*xy*_). Regarding the behaviour of *q* *m* *xy*, shown in the upper panels of both figures, in all cases it has a weak dependence on *z*_s_, displaying a rather flat profile except at the highest values of *z*_s_, where close contacts in mainly horizontal arrangements are very unlikely. These figures also evidence that *q* *m* *xy* is basically independent of the sampled values of applied field and grafting density. As we pointed out above only temperature, due to its interplay with the steric and dipole–dipole interactions, is a determinant for the value of *q* *m* *xy*. However, the behaviour of *S*_s_(*q* *m* *xy*), shown in the lower panels of Fig. 4(a) and (b), turns out to be quite more complex. Only for the case of very high temperature, *T* = 5, *S*_s_(*q* *m* *xy*) is basically independent of *z*_s_ for both grafting densities, evidencing that the thermal fluctuations tend to make the system more isotropic. As the temperature is decreased, the changes in the profile of *S*_s_(*q* *m* *xy*) correspond to a significant growth of the number of horizontally touching particles, as was initially observed in the overall measures of *S*(*q*_*xy*_). Importantly, *S*_s_(*q* *m* *xy*) also evidences that this growth is mainly happening at intermediate values of *z*_s_. In particular, for *σ* = 0.040 such an increase is observed between *z*_s_ = 2.5 and *z*_s_ = 4.5. At the lowest temperature and the same grafting density, the profile shows two maxima: a pronounced maximum at *z*_s_ = 4 and a weaker one at *z*_s_ = 2.5. For *σ* = 0.111 instead, the maximum at the lower position is shifted to *z*_s_ = 3 and is higher than the one at *z*_s_ = 4. The external field has an impact on *S*_s_(*q* *m* *xy*) qualitatively analogous to the temperature, flattening its profile and decreasing its values, but in this case as a consequence of the vertical straightening of the filaments induced by the field.^37^ The fact that the growth of horizontal close contacts with decreasing *T* and *H* is stronger at intermediate *z*_s_ can also be explained by the way that strongly bended filaments contribute to *S*_s_(*q* *xy* *m*). To better illustrate this, in Fig. 3(a) we sketch a strongly bended filament (with a darker colour) that touches with its free end a particle that belongs to a neighbouring filament and is located near the grafting surface. In this example, it can be seen that up to four of its particles are found in a mainly horizontal arrangement within the slice *z*_s_ = 4, thus contributing with three pairs to the counting of horizontal close contacts in that slice. Since at low *T* the filaments tend to adopt configurations with smooth backbones,^37^ one can assume that any strongly bended filament that keeps the free end close to the grafting surface has a segment whose particles adopt a mainly horizontal arrangement. For the filament length sampled here, most of such segments will be found within the region 2 ⪅ *z*_s_ ⪅ 5.

At this point we have shown that, in comparison to conventional overall scattering, slice-by-slice scattering measurements can give a much better picture of the internal structure of magnetic filament brushes. We provided interpretations of the main features of such measurements on the basis of our previous studies, that were mainly focused on the characterisation of the topology of the clusters and whole networks of particles. However, the interpretation of the finest details of slice-by-slice scattering data requires an analysis of the simulation results focused on the formation of close contact pairs. In addition, this approach can overcome the limitations of the scattering measurements and provide a deeper insight into the internal structure of the system. In the next section we perform such analysis by means of a set of new structural parameters.

### 3.2 Internal structure

In the following we address two main points. First, we will explain the origin of the double maxima observed in the distribution of *S*_s_(*q* *m* *xy*) at low *T* and zero field. We will show that this feature is essentially determined by the self-assembly behaviour of the filament free ends. Second, we will analyse the mechanisms of formation of close contact pairs, including features that cannot be resolved by slice-to-slice scattering measurements.

#### 3.2.1 Density profiles of free ends

**Fig. 5:**
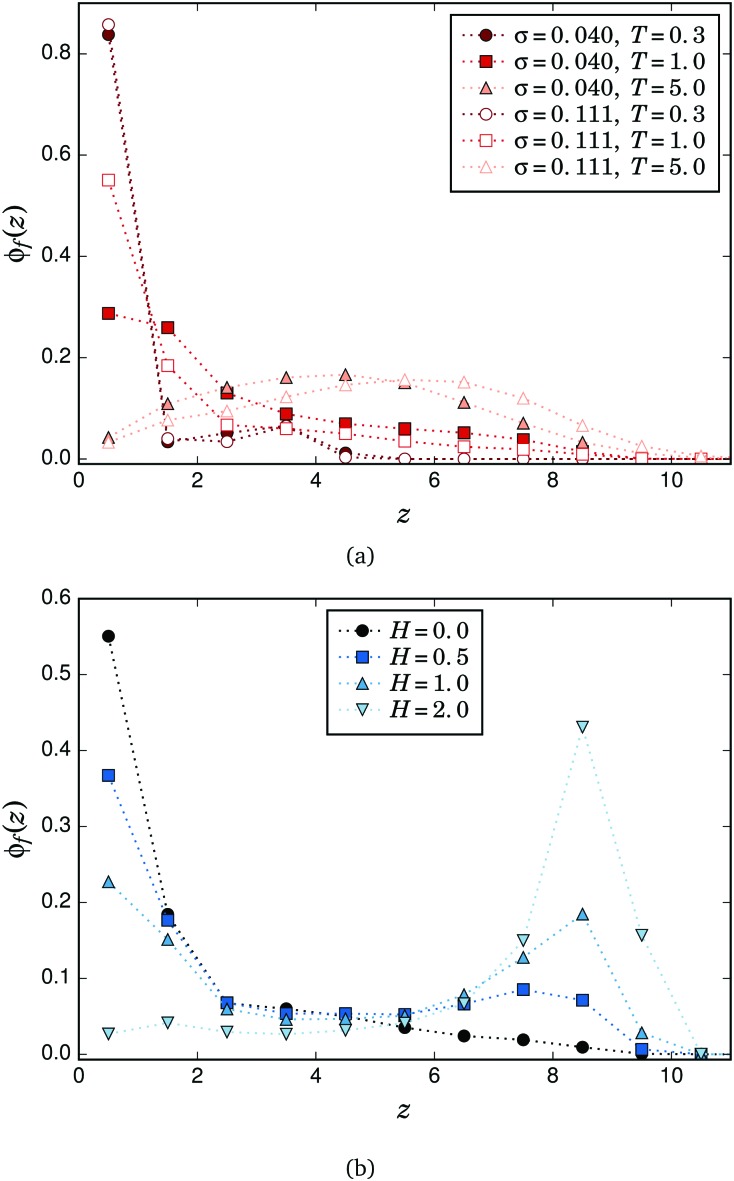
Evolution of the density profile of the free ends. (a) For different *T* and both grafting densities. (b) As a function of the applied field, *T* = 1 and *σ* = 0.111.

In order to find the origin of the two maxima observed in the profile of *S*_s_(*q* *m* *xy*), we calculated the vertical density profiles of the filament free ends, *φ*_f_(*z*). The effects of temperature and grafting density on *φ*_f_(*z*) can be seen in Fig. 5(a). For the lowest *T*, the profiles for both values of *σ* are very similar: first, they have a clear maximum at the position of close contact with the grafting surface, *z* ∼ 0.5, that corresponds to strongly bended filaments whose free ends touch the grafted ends of neighbouring filaments; second, there is a small broad maximum at *z* ∼ 3 that corresponds to lower but still strong filament bendings, so that their free ends do not touch the grafting surface but are still relatively close to it. The darker filament sketched in Fig. 3(a) is also an example of the latter type of configuration, in this case with its free end located within the slice *z*_s_ = 2. In the following, we will use ‘fully bended’ and ‘almost fully bended’ filaments to refer to those configurations that allow their free ends to touch or to be relatively close to the grafting surface, respectively. This distinction is important because the vertical position of the mainly horizontal part of a strongly bended filament depends on the vertical position of its free end: roughly, the lower the latter, the lower will be the former. Therefore, the two maxima in the profile of *S*_s_(*q* *m* *xy*) at low *T* correspond to the horizontal segments associated with the two preferred vertical positions of the filament free ends observed in *φ*_f_(*z*). The slight shift in the position of the first maximum with grafting density (from *z*_s_ ∼ 2.5 for *σ* = 0.040 to *z*_s_ ∼ 3 for *σ* = 0.111) is due to the different horizontal extension that fully bended filaments of a given length have to achieve in order to reach with their free ends the grafting positions of near neighbouring chains: the lowest *σ* imposes a larger distance between grafting points, so the filaments need a higher horizontal extension and their horizontal segments are found at lower *z*_s_. A less subtle difference in the comparison of grafting densities is the notably large value of the maximum of *S*_s_(*q* *m* *xy*) observed at *z*_s_ = 4 for *σ* = 0.040 and *T* = 0.35. This pronounced maximum implies a high relative number of horizontally touching particle pairs within the slice. Since the local density of particles at such *T* and *σ* is very low or zero for heights above *z*_s_ ⪆ 4,^36^ this means that most of the few particles found in that slice actually belong to horizontal segments of almost fully bended filaments. Despite the local density at *z*_s_ = 4 being very similar for both *σ*, at the higher grafting density the filaments are in general more entangled and disordered,^37^ and the fraction of particles in horizontal segments at that height is lower. As the temperature grows, the maximum of *φ*_f_(*z*) at *z* ∼ 0.5 tends to vanish, showing how the thermal fluctuations hinder the full bending of the filaments and the touching of their free ends to the grafted ones of their neighbours. This corresponds to the decreasing and flattening of the distribution of *S*_s_(*q* *m* *xy*) values shown in Fig. 4(a). At very high *T*, the distribution becomes rather broad, spanning up to the contour length of the filaments and showing a low maximum in the central region, which corresponds to a rather isotropic internal brush structure. A similar analysis can be performed for the effects of the external field. Fig. 5(b) shows the density profiles obtained for the free ends of a brush with *σ* = 0.111 when exposed to different field strengths at *T* = 1. At low *H*, the absolute maximum at *z* ∼ 0.5 corresponding to fully bended filaments can also be observed, whereas no maximum corresponding to almost fully bended configurations can be distinguished for any nonzero sampled field. This explains the absence of a clear second maximum in *S*_s_(*q* *m* *xy*) at nonzero fields, as shown in Fig. 4(b). As *H* increases, the absolute maximum in *φ*_f_(*z*) moves from the grafting height to almost the contour length of the filaments, showing the vertical straightening of the chains with the field. As we already pointed out above, this straightening is the reason for the flattening and strong decrease of *S*_s_(*q* *m* *xy*) with field.

#### 3.2.2 Connections

The discussion provided so far shows that the main contribution to the horizontal slice-by-slice scattering comes from segments of crosslinked particles belonging to the same filament, but this contribution is actually controlled by the self-assembly behaviour of the free ends. Besides this, we still need to obtain a proper understanding of the self-assembly between any pair of particles belonging to different filaments. In particular, we need to determine the main driving mechanism—either dipole–dipole interactions or thermal fluctuations—for the formation of touching particle pairs under given conditions. In order to study these aspects, we need a general criterion to consider two particles to form a touching pair, or connection. This criterion should necessarily involve the characteristic distance between touching particles in the system, which mainly depends on *T*. Besides that, one can use an interaction energy threshold in order to ignore entropically driven connections. Since scattering measurements are unable to discriminate the entropic contributions, the latter criterion is less suitable for the interpretation of structure factors. In this work, however, we decided to compare both a simple distance threshold criterion and a combination of energy plus distance threshold criteria. In this way, we are able to determine the fraction of energetically bound pairs among those detected by scattering. In our system, it is also important to separate the contribution of connections due to permanent crosslinking from those formed spontaneously. The permanently bonded connections, together with the energy driven self-assembled ones, are responsible for the viscoelastic and magnetic properties of the brush. Finally, we also investigate the pair formation in a nonmagnetic brush in order to trace back the role of magnetic dipolar interactions in the structural behaviour of the system.

**Fig. 6:**
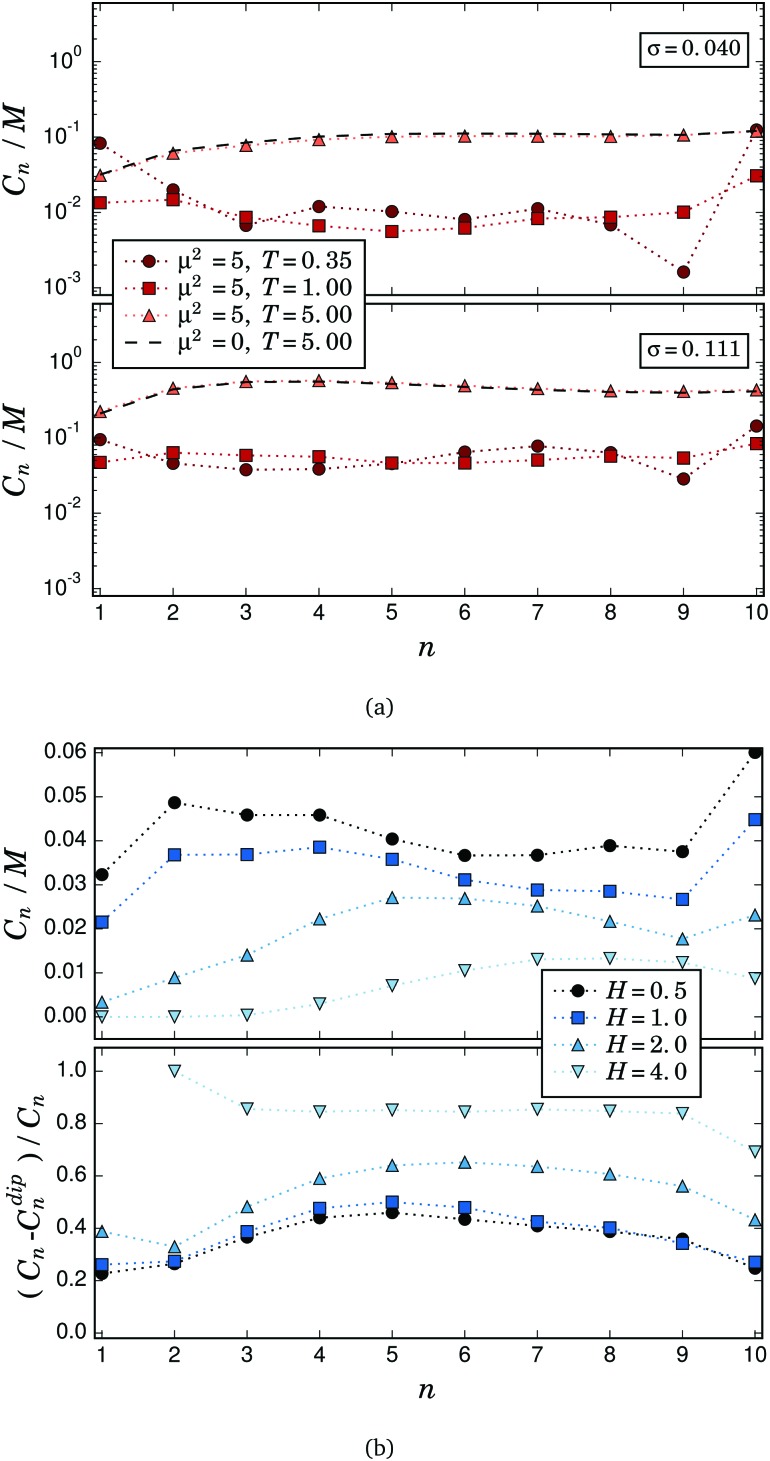
Connections: (a) for different temperatures and both grafting densities (a semilogarithmic scale is used due to the large differences in values); (b) for different field strengths, *T* = 1 and *σ* = 0.111.

For the simple distance criterion, we consider two particles to be connected if they belong to different filaments and their centre-to-centre distance satisfies *r*_*ij*_ ≤ max(*r*_cut_,*b*_max_), where *r*_cut_ is the cutoff distance for the soft core potential (2) and *b*_max_ is the maximum bond length between crosslinked particles. We number the particles in each filament from 1 to 10, starting with the grafted one. In Fig. 6 we plot the average number of connections found with this criterion for each *n*-th particle along the filaments, *C*_*n*_, divided by the total number of particles in the system, *M*. Fig. 6(a) shows *C*_*n*_/*M* for several selected values of *T* and both grafting densities. As one can see, for *σ* = 0.040 the particles corresponding to the grafted and free ends are the most connected at low temperatures. This is consistent with the low temperature structure dominated by fully bended filaments that was discussed above when analysing the height distribution of free ends and the scattering response. With growing *T*, the distribution of connections along the filaments flattens and finally undergoes a qualitative change: at *T* = 5 almost all the particles along the filament except for the grafted ones have basically the same number of connections. An increase in the number of connections with the temperature can also be observed. The grafting density does not affect qualitatively this behaviour, but shifts the curves towards higher values. We also performed the same analysis using the combined distance and energy threshold criteria. For the latter, we impose that the dipole–dipole pair energy has to be negative in order to consider a connection to be energy driven. The results (not shown) exhibit the same qualitative behaviour for any *n*, *T* and *σ*, and only a quantitative difference that grows with *T* can be observed: the combined criteria give a lower amount of connections, ranging approximately from 8% for *T* = 0.35 to 50% for *T* = 5. In other words, magnetic dipolar interactions, if strong enough, define qualitatively the profile of both energetic and entropic connections. Moreover, at low temperatures most connections are energy driven, whereas for high *T* thermal fluctuations become dominant. To prove the latter, we also show in Fig. 6(a) the profile of the connections corresponding to an analogous nonmagnetic brush (*μ* ^2^ = 0) for *T* = 5 and both grafting densities. It can be observed that these curves are almost identical to the ones of the magnetic brush at the same *T* and *σ*. In the upper panel of Fig. 6(b) we present the results obtained for *C*_*n*_/*M*, using the distance criterion, for *σ* = 0.111, *T* = 1 and different external magnetic fields. As expected from the discussion on the scattering properties presented above, the connections tend to disappear with a growing field, an effect that is much stronger for the lower grafting density (not shown). Interestingly, the field strongly hinders the connections of the particles closer to the grafted end of the filaments, whereas for *n* > 4 the decrease in *C*_*n*_ with field is less pronounced. This can be explained by the higher structural entropy of the filament free ends. Besides that, the free ends keep a relatively high connectivity at low fields: the curves corresponding to *H* = 0.5 and *H* = 1.0 show an absolute maximum at *n* = 10. Comparing it to Fig. 5(b), we can conclude that these connections are located at *z* ∼ 8. In order to check whether the connections established under field are energetically or entropically driven, we also plot in the lower panel of Fig. 6(b) the relative difference between the average number of connections obtained for the distance criterion, *C*_*n*_, and the ones calculated with the distance–energy criterion, *C*dip*n*. This quantity is a measure of the relative fraction of entropic connections in the system, so that it reaches unity when all the connections are entropy driven. From this parameter one can conclude that, while the total amount of connections decreases with field, the fraction of those driven by entropy grows, becoming practically the only type that remains at high fields. The only few energy driven connections that remain are predominantly between the middle particles of the filaments, forming configurations similar to the X-junctions observed in self-assembled chains of free magnetic particles.^70^ According to the scattering measurements shown in Fig. 4(b), the position of these connections is between *z*_s_ = 2 and *z*_s_ = 5.

In general, the analysis of *C*_*n*_ confirms the physical explanation provided in the sections above concerning the behaviour of *q* *m* *xy* and *S*_s_(*q* *m* *xy*). The last step to achieve a detailed understanding of the internal structure of the filament brush is to identify the particles more involved in the formation of touching pairs according to their position along the filaments. This point is addressed in the next section.

#### 3.2.3 Connectivity maps

**Fig. 7:**
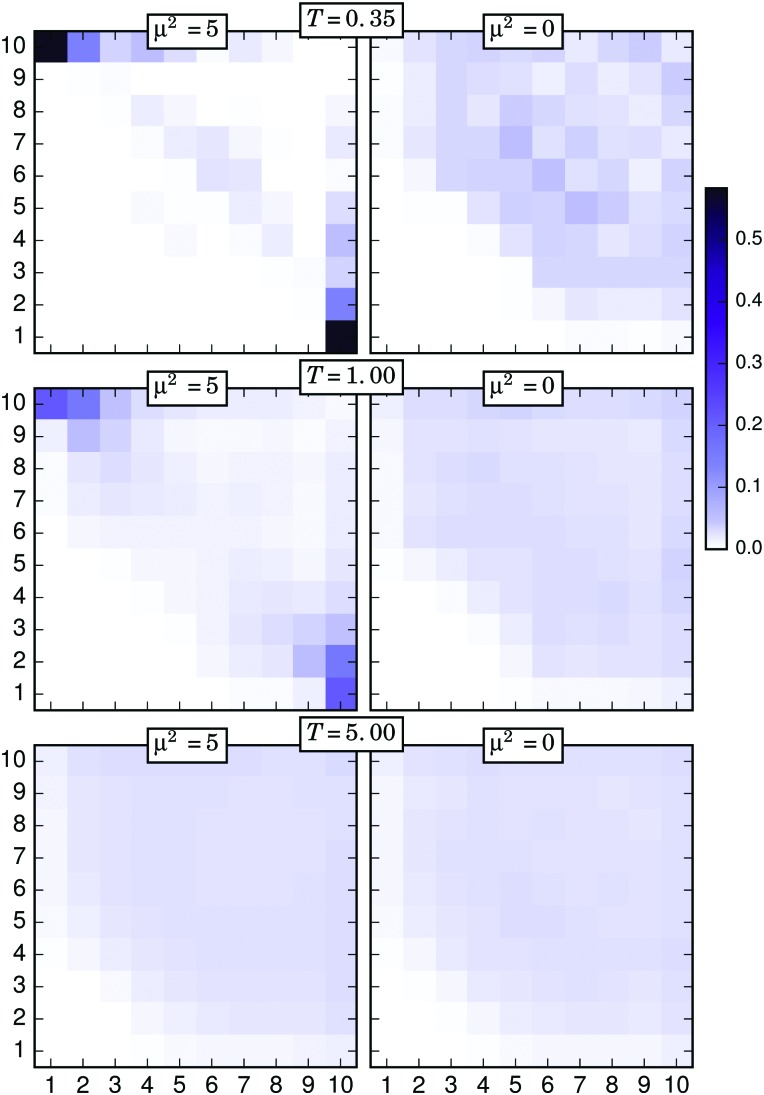
Connectivity probability maps for different selected temperatures and *σ* = 0.040, obtained with the simple distance threshold criterion. Left column corresponds to a magnetic filament brush, right column to an analogous nonmagnetic brush.

**Fig. 8:**
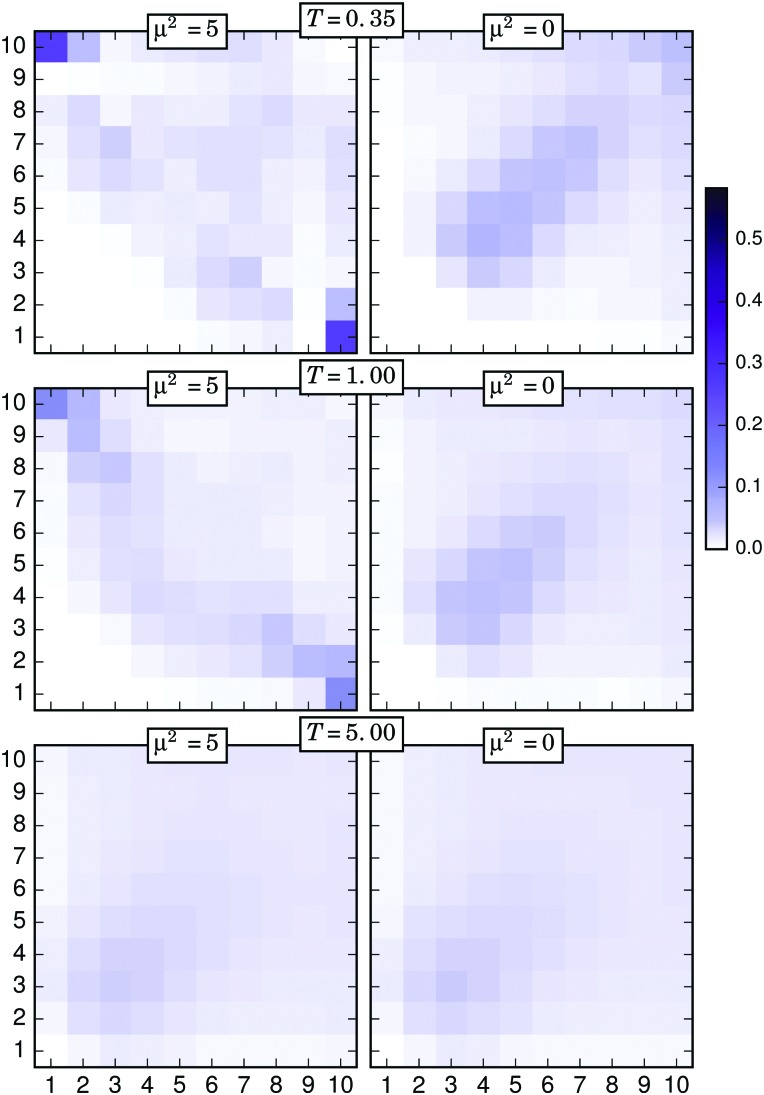
Connectivity probability maps for the same selected temperatures used in Fig. 7 and *σ* = 0.111, also obtained with the simple distance threshold criterion. Left column corresponds to the magnetic filament brush, right column to the nonmagnetic one.

**Fig. 9:**
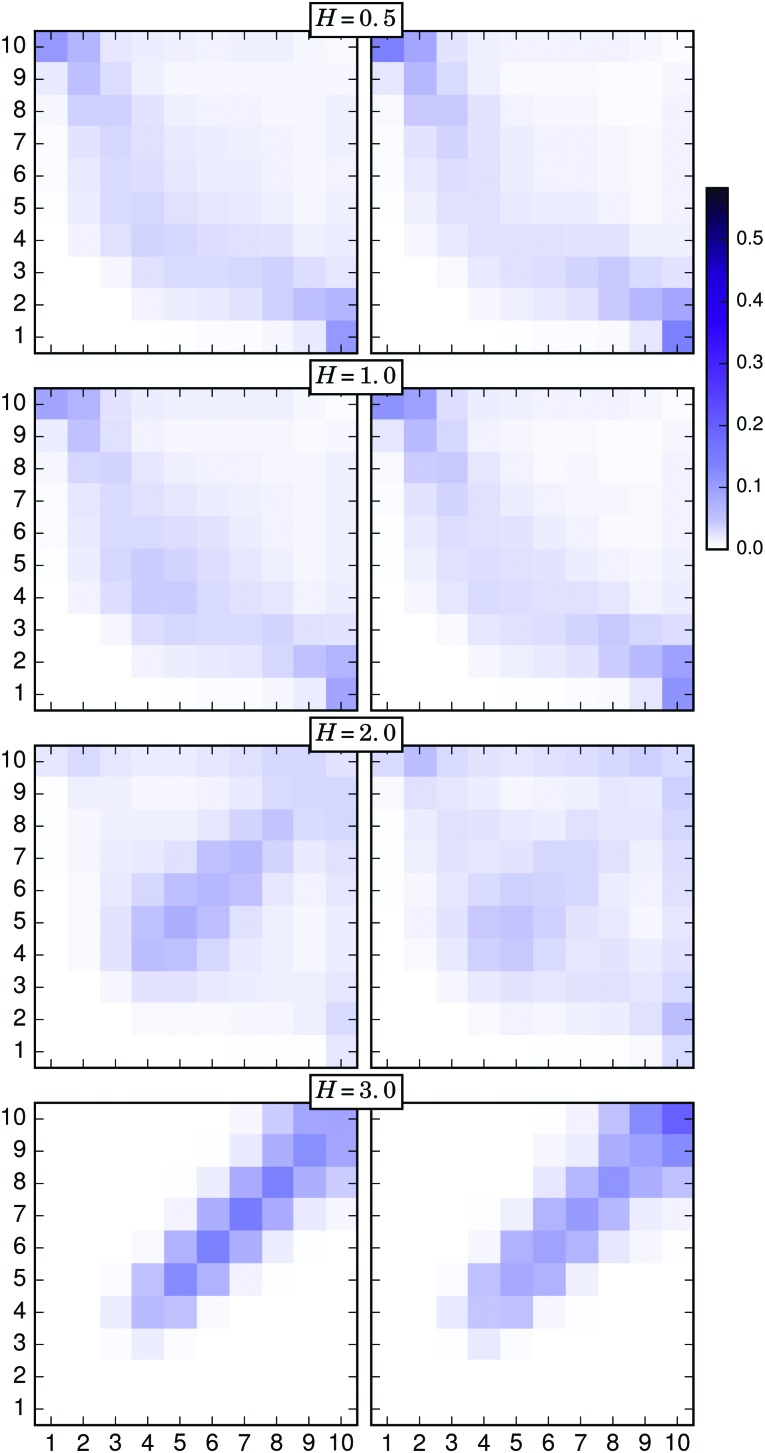
Connectivity probability maps for different external field strengths and *σ* = 0.111. Left column: Results obtained with the simple distance threshold criterion. Right column: Results corresponding to the combined distance–energy criterion.

The nonuniform distribution of connections discussed above evidences the importance of the position of a particle along its filament in order to form touching pairs. In order to analyse this characteristic, we calculated the probability of two connected particles to be located at positions *i* and *j* of their respective filaments. The results obtained from this calculation can be represented as two-dimensional, symmetric probability arrays with dimensions *N* × *N*. Fig. 7–9 include the graphical representation of such probability arrays obtained under different conditions. In each plot, or connectivity map, the axes number the discrete positions *i* and *j* of each particle of the touching pair along its respective filament, following the same criterion introduced above—1 for the grafted particle, 10 for the free end—whereas the probability, normalised for one half of the symmetric array, is represented with a colour scale.

The connectivity map obtained using the simple distance criterion for *T* = 0.35 and *σ* = 0.040 is shown in the upper left corner of Fig. 7. One can easily see that, under these conditions, the connections are restricted to only a few different types of pairs. Most of the connections involve one free end, with the (1,10) pair—corresponding to the connection between grafted and free ends—being the most probable case by a large margin. Connectivity of free ends also shows a local maximum with the 4-th and 7-th particles, which is consistent with the case of almost fully bended filaments connecting their free ends to central parts of other strongly bended neighbours. Some combinations between upper middle particles—that can be identified as X-junctions—are also observed. This restricted behaviour changes with growing *T*, as it can be seen following the left column of the figure from top to bottom. For *T* = 5, the maxima corresponding to the pairs of free and grafted ends disappear and the probability distribution becomes rather homogeneous, with the only absence of those combinations forbidden by the geometrical constraints. To clarify if the connections are energy driven, in the right column of Fig. 7 we plot the connectivity maps of a nonmagnetic brush under the same conditions. A striking difference can be observed for the connections of (1,10) type: in a nonmagnetic brush, free ends never connect to grafted ones. In other words, a fully bended filament is always an energy driven configuration. The difference between the right and the left column tends to fade off as the temperature grows. An analogous qualitative behaviour is found for the case *σ* = 0.111, that is shown in Fig. 8. The main difference with respect to the former case comes from the higher degree of filament entanglements led by the higher grafting density, a fact that is reflected in a less restrictive distribution of connection types at low *T*. Note that, independently from *σ*, particles at the 9-th position have a very low probability to participate in pairs formed at low *T*, but become rather active in nonmagnetic brushes or at high temperatures. Instead, both free ends of neighbouring filaments basically never form touching pairs at low temperatures, but easily connect if entropy dominates in the system.

The behaviour of the filament free ends is also a determinant for the structure of the brush under an applied external field. Fig. 9 shows the connectivity maps calculated for *σ* = 0.111 and different field strengths. In this case, the left column corresponds to the results obtained with the simple distance threshold criterion, whereas the right column includes the results obtained with the combined distance–energy criterion. At very low fields, independently of the applied criterion, the connection between free and grafted ends is still the most probable combination, but it quickly disappears as the field increases. Also for both criteria, the transition induced by a growing field to a structure of rather vertically straight filaments can be clearly observed. For such structures, X-junctions between pairs of particles located at very similar positions along the filaments become the only allowed possibility. Interestingly, it is in the distribution of this latter type of connection where the main qualitative difference between the results provided by each connectivity criterion can be observed: by comparing the left and right columns at high fields, it can be clearly seen that when all connectivity mechanisms are considered—*i.e.*, the simple distance criterion is used—the most probable type of connection is the (7,7) pair, whereas the most probable energy driven type of connection corresponds to both free ends. The latter is due to the fact that (10,10) connections allow a high alignment of the filament backbone—and with it, of the magnetic moments of the particles—to be maintained in the direction of the field and, at the same time, are the most entropically favorable.

## 4 Conclusions

In this work we studied the possibility of using scattering techniques to investigate the internal structure of magnetic nanoparticle filament brushes. By means of extensive computer simulations, we analysed the expected scattering response of the equilibrium configurations of this system, obtained for different temperatures, external fields perpendicular to the grafting surface and grafting densities. The features of the scattering measurements have been explained on the basis of the detailed internal structure of the filament brush, showing how computer simulations can assist the interpretation of the measurements of intensity profiles and structure factors.

Our results suggest that experimental measurements in thin slices parallel to the grafting surface are important for the proper analysis of the magnetic filament brush structure, due to the intrinsically strong inhomogeneity of this system along the axis perpendicular to the grafting surface. Additionally, we analysed the mechanisms of formation of touching particle pairs within the brush, which are responsible for the existence of the first local maximum in the profile of the structure factor. By comparing the distributions of the touching particle pairs defined by two different criteria—a simple distance threshold criterion and a combined distance–energy threshold criterion—we were able to distinguish between entropy and energy driven connections. We have shown that the total number of connections grows with temperature and decreases with field, but the entropy driven connections tend to become dominant as both *T* and *H* grow.

We also provided evidence on the important role that filament free ends have for the equilibrium behaviour of the brush, showing that the overall structural changes induced by temperature and external fields are associated with changes in the distribution of the free ends. In addition, we determined that, in contrast to particles located at any other position along the filaments, the connections established by the free ends tend to be energy driven in most cases, thus they play the main role in the self-assembly properties of the system.

Finally, our results point out a way to effectively select the distance from the grafting surface at which more particle pair connections can be found. We have shown that such properties can be controlled by means of the background temperature and/or the strength of the external field: with growing *T* and *H*, the height with a larger amount of connections increases, starting from a few particle diameters up to almost the filament's contour length. This result is of particular importance for microfluidics and rheological applications, as it points to the possibility of modifying the velocity profile of the flux through the magnetic filament brush by means of the external stimuli.
